# Supplementary material for: Cell type determination for cardiac differentiation occurs soon after seeding of human-induced pluripotent stem cells
Source: Genome Biol. 2022 Apr 5;23:90. doi: 10.1186/s13059-022-02654-6 (PMC8985385; doi:10.1186/s13059-022-02654-6)
Supplement: Supplementary file 1 — Additional file 1: Figures S1-6. [file 13059_2022_2654_MOESM1_ESM.pdf]

Fig S1

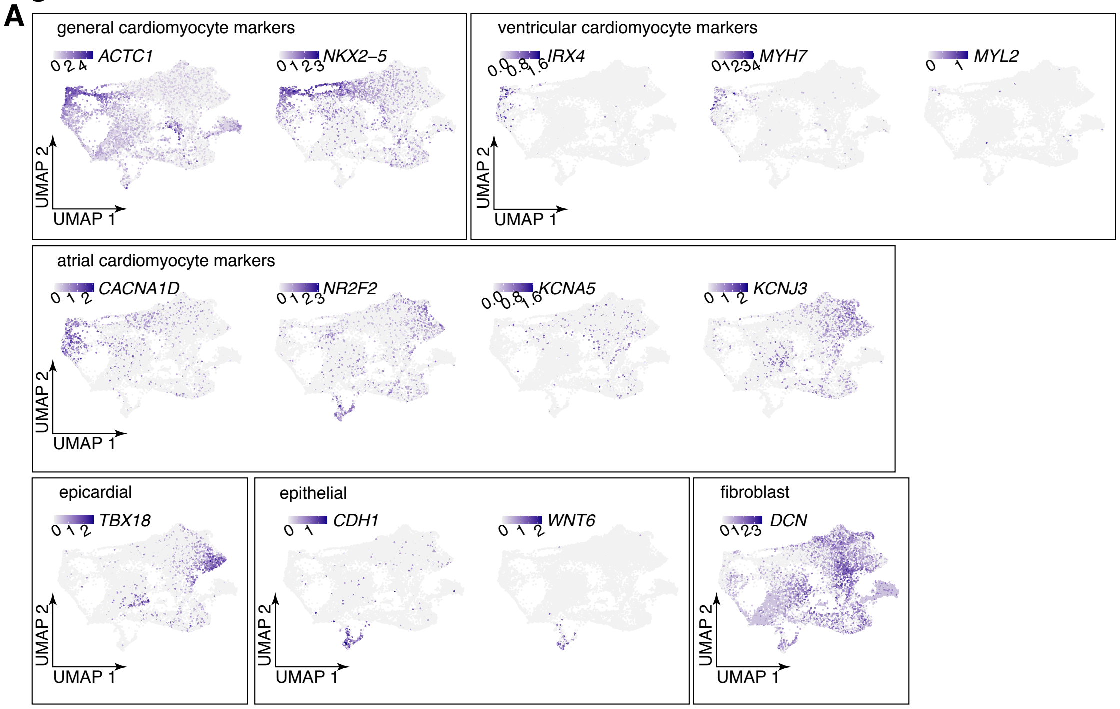

Fig S1. Localization of additional cell type markers following cardiac directed differentiation in day 14 differentiated cells. (A) Maintaining the organization provided by UMAP, we recolored each cell by its expression of a number of canonical cell type markers; some of these are also listed in the heatmap in Figure 1C while others are important in cardiac biology.

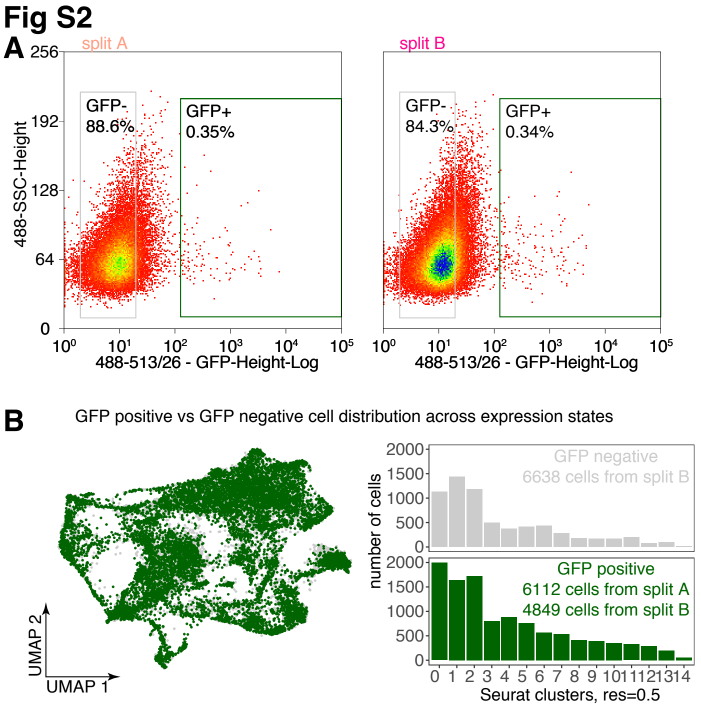

Fig S2. GFP positive and negative sorted cells are largely transcriptionally indistinguishable. (A) We sorted cells from both splits A and B by GFP expression using FACS. Dots represent individual cells. Plots were constructed using the final 50,000 sorted cells from each sample. (B) Maintaining the organization provided by UMAP, we plotted all sequenced GFP negative sorted cells in grey and all sequenced GFP positive sorted cells in green. Bar graphs demonstrating the distribution of GFP positive and negative cells across Seurat clusters, colored the same way. Note that we only sequenced a portion of the GFP negative population, taken from the sorted split B GFP negative cells.

Fig S3

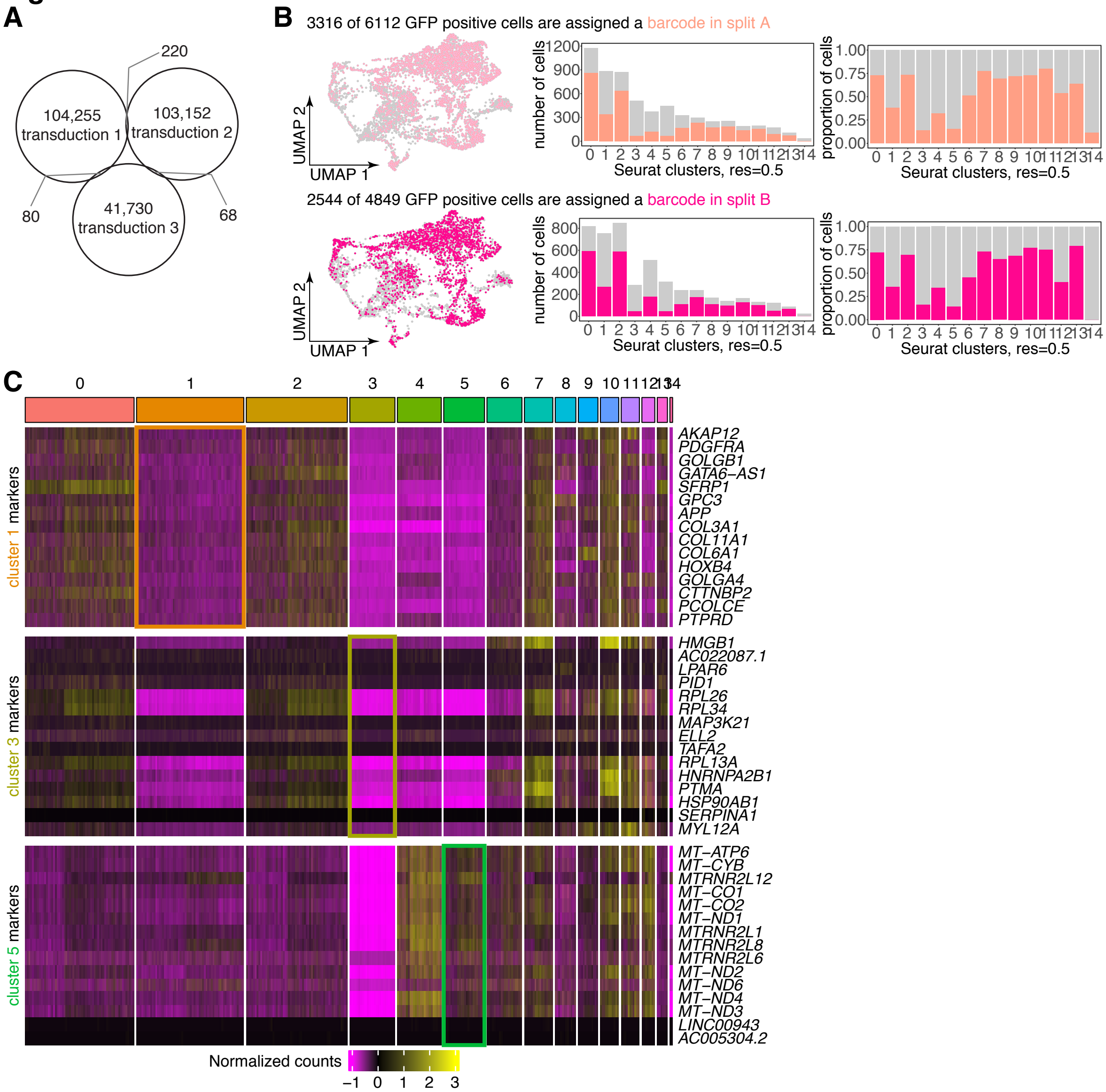

Fig S3. Barcode library complexity and cell distribution across Seurat clusters. (A) Overlap in barcodes recovered from 3 independent transductions as visualized using a Venn diagram, adapted from Goyal *et al.* 2021. Using these overlaps we estimate that we have over 45 million unique barcodes in our library (see Methods for details). (B) Maintaining the organization provided by UMAP, we recolored all GFP positive sorted cells in each differentiated split (A or B) profiled by 10X single-cell RNA sequencing (grey) and then recolored the cells for which a barcode was recovered in salmon (split A) or magenta (split B). Bar graphs demonstrating the raw cell distribution (left) and proportion (right) of barcoded cells across Seurat clusters in split A (salmon, top) or split B (magenta, bottom) as compared to GFP positive cells unable to be assigned a barcode (grey). (C) Heatmap showing normalized gene expression for the top 15 markers for Seurat clusters 1, 3, and 5 across all 17,599 cells. Mitochondrial markers are relatively overrepresented in cluster 5, whereas there are not good gene cluster markers for clusters 1 and 3.

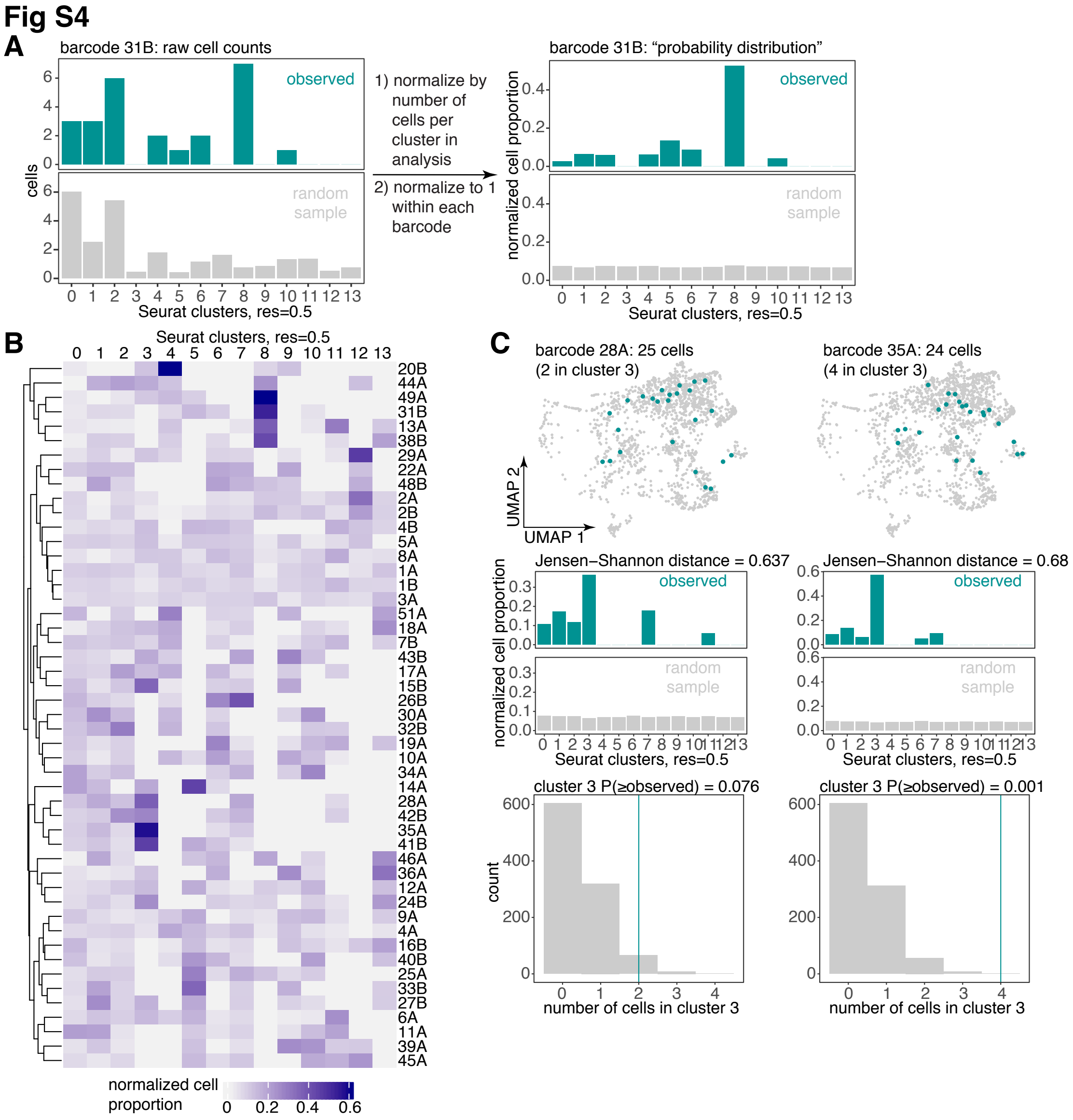

Fig S4. "Probability distribution" of barcoded clones across Seurat clusters. (A) Explanation of how we derived cluster probability distributions from raw cell cluster distribution for each barcoded clone, using barcode 31B as an example. (B) Heatmap demonstrating the normalized cell proportion across Seurat clusters for all barcodes in the analysis from Figure 2. Most clusters are associated with low normalized cell proportions (light purple) across barcodes, but clusters 4, 8, and 12 have more binary behavior (i.e. either high normalized cell proportion or very low normalized cell proportion). (C) Analysis determining whether barcode 28A and 35A cluster 3 overrepresentation as visualized in B is significant. Maintaining the organization provided by UMAP, we plotted all cells in the analysis (grey) and recolored cells corresponding to barcodes 28A (left) and 35A (right) in teal. Also for these barcodes, we plotted bar graphs for observed cluster probability distribution (teal) and the average random cluster probability distribution (grey). Finally, we plot histograms demonstrating the distribution of the number of cells that would land in cluster 3 by random chance as generated from 1000 random samplings of an matched number of cells in the analysis (i.e., sampling 25 cells for barcode 28A and 24 cells for barcode 35A, grey) as compared to the observed number of barcode 28A or 35A-labelled cells assigned to cluster 3 (teal vertical line). Cluster 3 behavior is statistically significant for some barcode clones (ex. barcode 35A), perhaps because few GFP positive cluster 3 clones are able to be assigned a barcode (see Supplementary Figure 3).

Fig S5

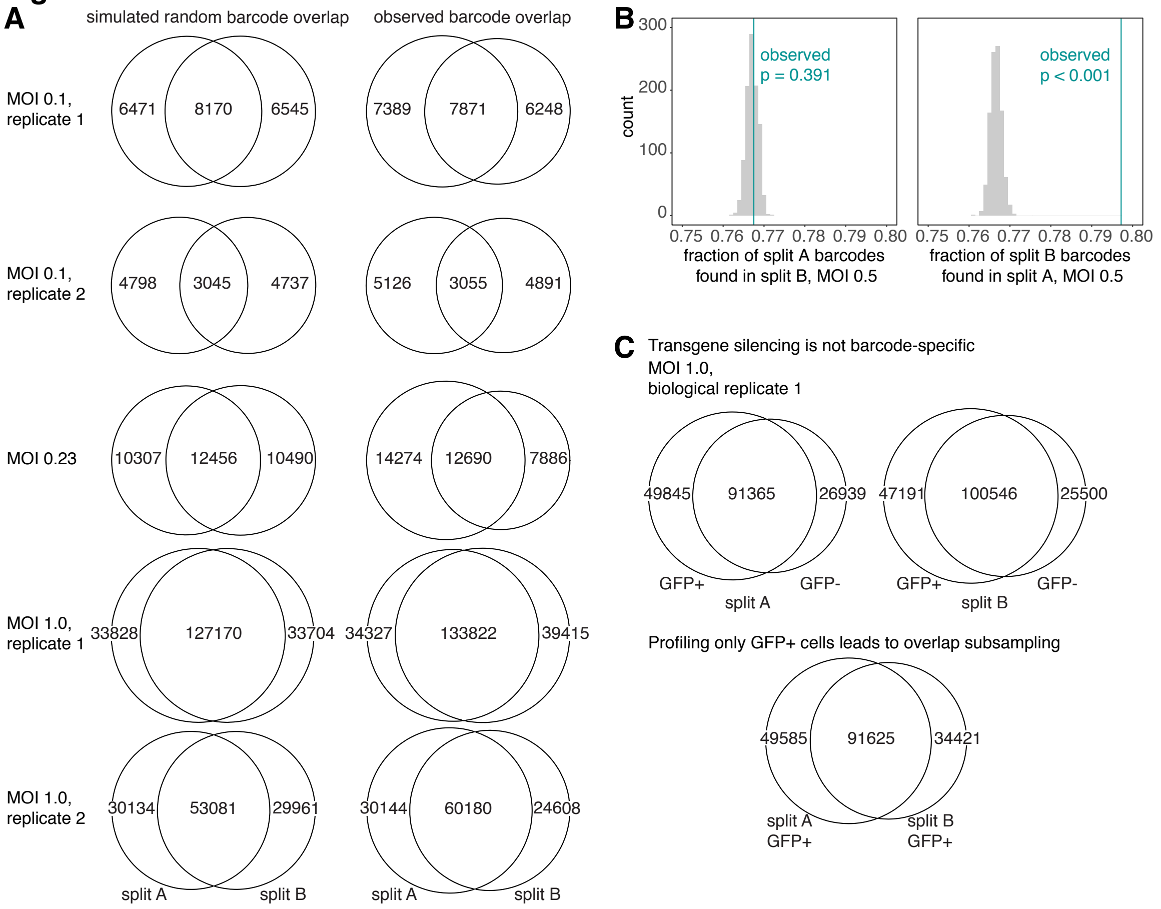

D

Barcode name	Cells in differentiation A	Cells in differentiation B
1	197	301
2	155	159
3	186	0
4	92	27
5	96	7
6	83	0
7	3	73
8	59	10
9	68	0
10	57	5
11	48	0
12	48	0
13	47	0
14	24	17
15	0	39
16	0	39
17	38	0
18	23	12
19	34	0
20	2	31

Fig S5. Neither survival nor transgene silencing are barcode-specific. (A) Comparison of the simulated random barcode overlap across splits with the observed barcode overlap from the splits differentiated from hiPS cells from all other transduction conditions (MOI 0.1, MOI 0.23, MOI 1.0). (B) Statistical analysis associated with the fraction of overlapping barcodes found after differentiation of cells transduced with an MOI of ~0.5 (Figure 3B). We found that there was no significant difference in overlap for split A. While we did find a significant difference in overlap for split B, the effect size of this difference was such that it may have stemmed from technical variation in barcode recovery and was deemed unlikely to be biologically relevant. (C) Venn diagrams demonstrating the overlap in barcodes found in sorted GFP positive and GFP negative subpopulations following differentiation for each split from cells initially transduced at an MOI of ~1.0. Profound overlap between GFP positive and GFP negative subpopulations suggests that GFP transgene silencing does not occur in an integration-specific or clone-specific manner. (D) Top 20 barcode clones comprising the most total cells across both splits, noting how many were recovered from each split. Pink (salmon for split A, magenta for split B) backgrounds associated with the barcodes that are represented on UMAP in Figure 3C.

Fig S6

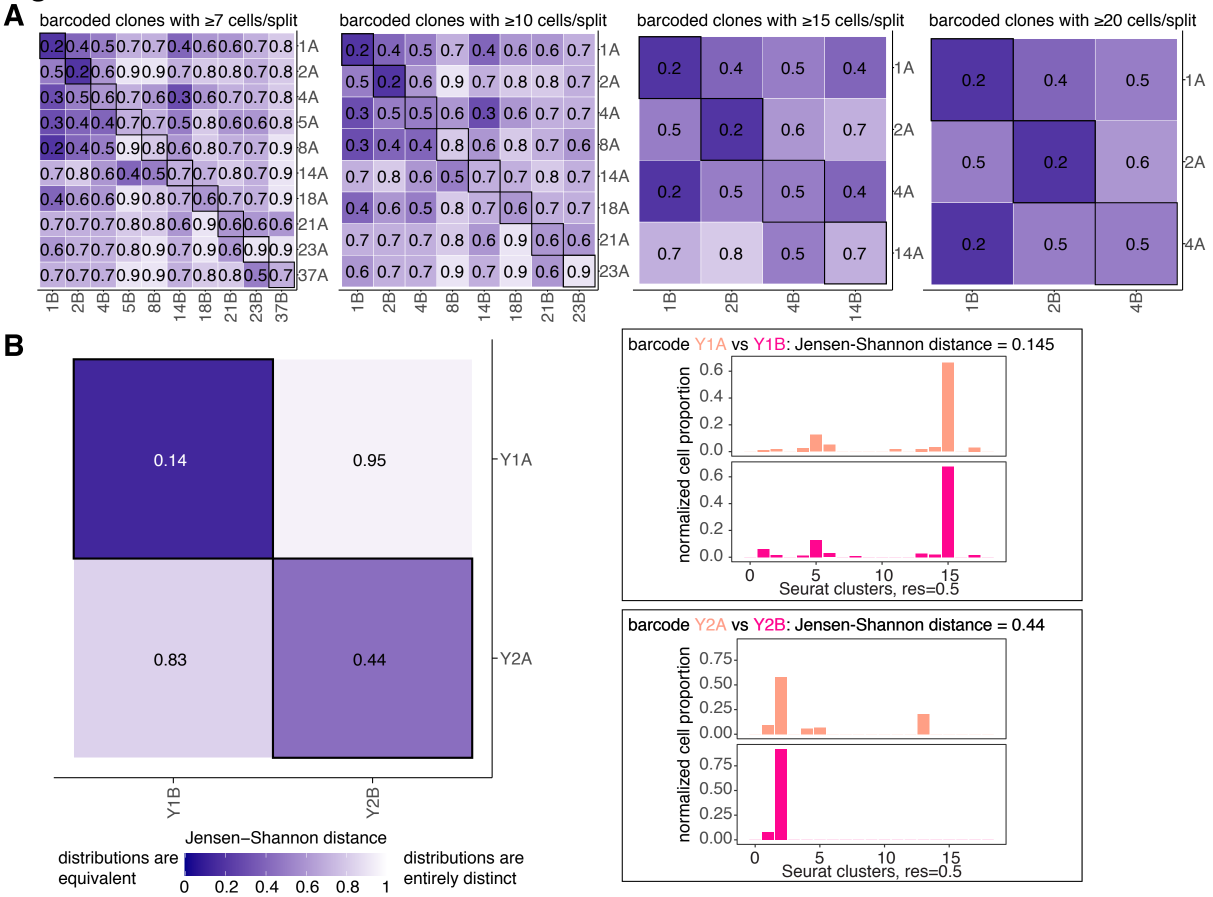

Fig S6. Jensen-Shannon distance captures known heritable predetermination of expression state. (A) Heatmap of pairwise Jensen-Shannon distances (smaller distance is darker in color) between split A and B cells associated with barcode clones with (from left to right) at least 7, 10, 15, or 20 cels per split. As demonstrated in Figure 4B, separated clones sharing a barcode (bolded outline along the diagonal) had Jensen-Shannon distances in the same range as clones labeled by distinct barcodes (off the diagonal). (B) Heatmap of pairwise Jensen-Shannon distances between cells associated with 2 barcodes in split A and the cells associated with the same 2 barcodes in split B from a dataset previously shown to have heritable predetermination of the final cell state (vemurafenib-treated melanoma cells). Separated clones sharing a barcode (bolded outline along the diagonal) had much smaller Jensen-Shannon distances than clones labeled by distinct barcodes (off the diagonal), demonstrating their similarity. This similarity is also visible in the comparison for each barcode of bar graphs for observed cluster probability distribution in split A (salmon) and the observed cluster probability distribution in split B (magenta). Cluster probability distributions are visually similar between separated clones and visually distinct across cells labeled by different barcodes.
